# How to measure intraocular pressure: Schiötz tonometry

**Published:** 2008-06

**Authors:** Sue Stevens

**Affiliations:** Nurse Advisor to the *Community Eye Health Journal*, International Centre for Eye Health, London School of Hygiene and Tropical Medicine, Keppel Street, London WC1E 7HT, UK.

**Figure:**
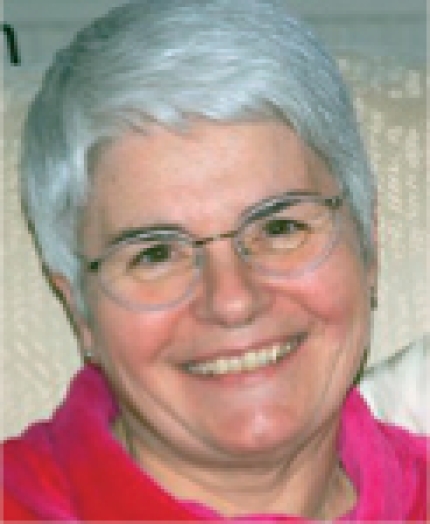


If glaucoma is diagnosed early, treatment can then be given that may preserve vision. Although raised intraocular pressure (IOP) is not the only sign of glaucoma, the IOP should be checked routinely on all adults attending eye care facilities. Applanation tonometry (as described in Issue 64, December 2007) is the most accurate method to measure IOP, but Schiötz tonometry is also a useful screening test. If Schiötz tonometry reveals a high IOP, this result should be checked and confirmed by applanation tonometry and the patient referred to the senior clinician at the eye clinic.

**You will need** (Figure 1)

**Figure 1:**
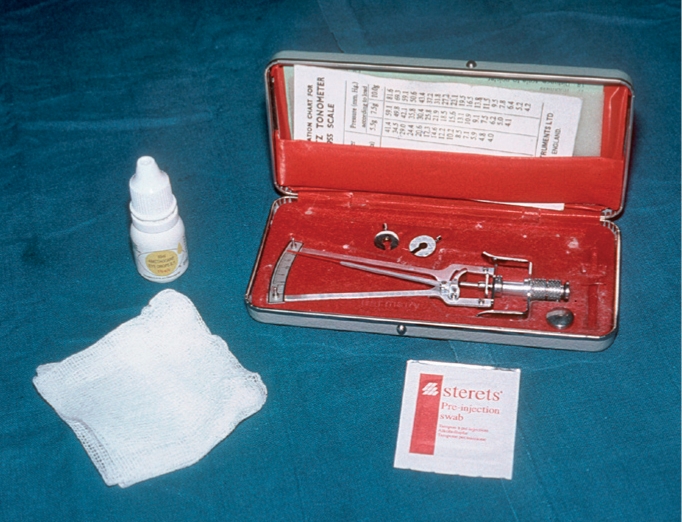

- Schiötz tonometer, weights, and scale card
- local anaesthetic drops
- clean cotton wool or gauze swabs
- isopropyl alcohol 70 per cent (methylated spirit) or impregnated ‘Mediswabs’.

## Preparation

- Test the tonometer using the spherical mould in the box and the 5.5 g weight. The pointer should immediately reach the ‘O’ marking (see Figure 2).
  **Figure 2:**
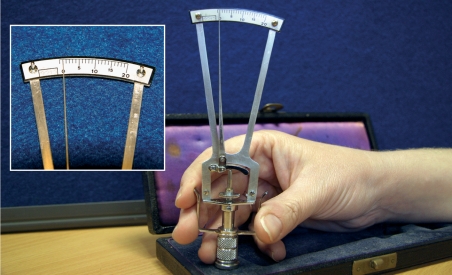

- Clean the plunger and disc of the tonometer with a gauze swab (or cotton wool) and the methylated spirit (or a Mediswab). Wipe dry with a clean dry gauze swab (or cotton wool).
- Lie the patient flat with his or her head supported on a pillow.

## Method

- Wash and dry your hands.
- Position yourself correctly: stand upright, behind the head of the patient, with your hands level with the patient's head. Note the health worker's good posture in Figure 3 and the awkward position of the health worker in Figure 4. Bad posture can affect the tonometry reading.
  **Figure 3:**
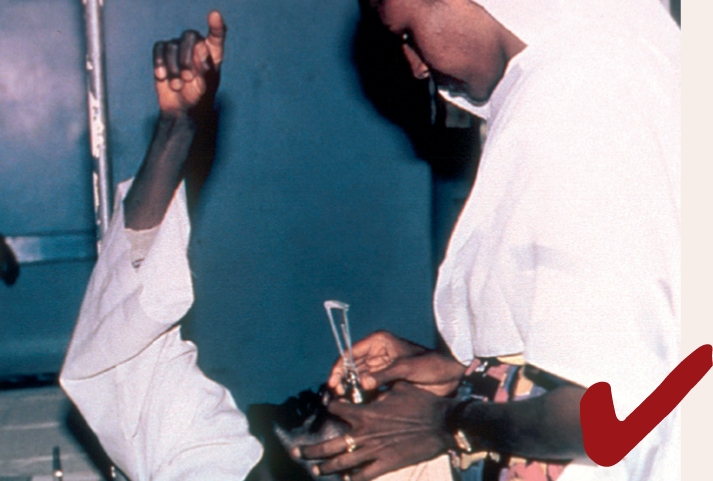
  **Figure 4:**
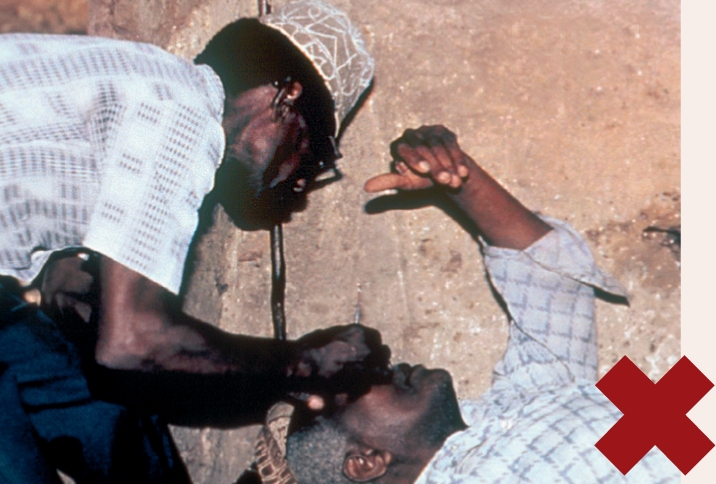
- Instil local anaesthetic eye drops and wait about 30 seconds.
- Ask the patient to look at a fixed object (the patient's own thumb or finger held directly in front of his or her eyes may work) and to keep absolutely still.
- With the thumb and index finger of one hand, gently hold open the patient's eyelids, taking care not to put any pressure on the eye (see Figure 5).
  **Figure 5:**
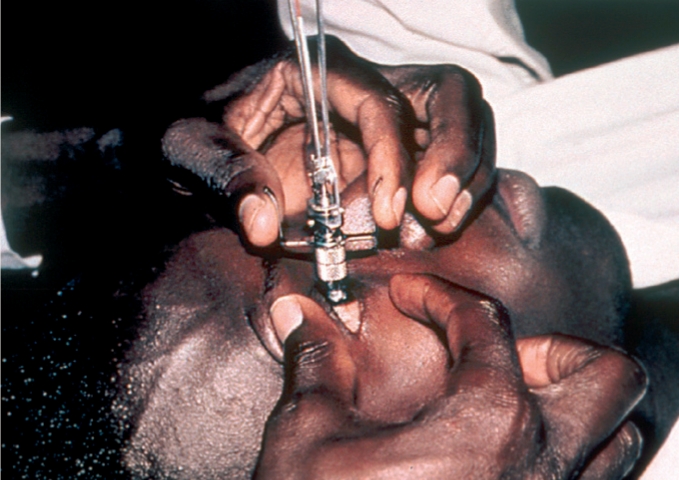
- With the other hand, hold the tonometer (with the 5.5 g weight) between the thumb and index finger and place the plunger on the central cornea (see Figure 5).
- Allow the disc to lower gently onto the corneal surface.
- Note the scale reading.
- If the scale reading is ‘2’ or less, remove the tonometer, replace the 5 g weight with the 7.5 g weight, and repeat the procedure.
- Note the scale reading again and remove the tonometer.
- Tell the patient not to rub the eye - the anaesthetic will last for about five minutes.
- Clean and dry the tonometer head.
- Repeat the whole procedure for the other eye.
- Clean and dry the tonometer again and store it safely in the box.
- Using the scale card, convert the noted scale readings and record the IOP in the patient's records.

